# Prognostic significance of the TREK-1 K2P potassium channels in prostate cancer

**DOI:** 10.18632/oncotarget.3782

**Published:** 2015-04-21

**Authors:** Gui-Ming Zhang, Fang-Ning Wan, Xiao-Jian Qin, Da-Long Cao, Hai-Liang Zhang, Yao Zhu, Bo Dai, Guo-Hai Shi, Ding-Wei Ye

**Affiliations:** ^1^ Department of Urology, Fudan University Shanghai Cancer Center, Shanghai, China; ^2^ Department of Oncology, Shanghai Medical College, Fudan University, Shanghai, China

**Keywords:** TREK-1, prostate cancer, biochemical recurrence, cell proliferation, cell cycle

## Abstract

**Background:**

*TREK-1* channels belong to the two-pore domain potassium channel superfamily and play an important role in central nervous system diseases. However, few studies have examined their role in carcinogenesis.

**Methods:**

In this study, we assessed the expression of TREK-1 in 100 prostate cancer (PCa) tissues using immunohistochemistry and further analyzed its clinicopathological significance. Next, cell proliferation and cell cycle analysis were carried out on human PCa PC-3 cell lines where *TREK-1* was stably knockdown.

**Results:**

We found that compared with normal prostate tissues, PCa tissues showed overexpressed TREK-1 levels and TREK-1 levels were positively associated with Gleason score and T staging. High level of TREK-1 expression was related to shorter castration resistance free survival (CRFS). Furthermore, knockdown of *TREK-1* significantly inhibited PCa cell proliferation *in vitro* and *in vivo*, and induced a G1/S cell cycle arrest.

**Conclusion:**

Our results suggest that TREK-1 might be a biomarker in CRFS judgment of PCa, as well as a potential therapeutic target.

## INTRODUCTION

Prostate cancer (PCa) screening has resulted in a dramatic reduction in advanced disease and disease-specific mortality [1]. Despite significant efforts in early diagnosis and treatment, many patients eventually relapse. Additionally, the mechanisms responsible for PCa carcinogenesis are not yet entirely understood. Therefore, it is critical to better understand the cell biology of PCa to identify novel molecular targets for therapy.

One potential set of targets are the potassium channels, which are transmembrane proteins that can selectively facilitate the permeation of potassium between extracellular and intracellular environments. Aberrant expression of potassium channels has been described in many cancers, such as colorectal cancer, breast cancer and acute myeloid leukemia [2–4]. Blockade of potassium channels has been demonstrated to inhibit malignant cell proliferation in small cell lung cancer, breast cancer and melanoma [5–7], suggesting new therapeutic potential.

As an important family of potassium channels, two-pore domain potassium channels (K_2p_) were identified as key players in a series of physiological and pathological processes, such as neuroprotection, cardiac activity modulation, anesthesia, depression and cancer [8–12]. Park et al. observed that the TREK-2 channel was present in bladder cancer cell lines and contributed to cell cycle-dependent growth [13]. TASK-3 was found to impact cell survival of glioma [14]. The present study was undertaken to detect the expression of TREK-1, another K_2p_ channel, in PCa tissues and explore its association with clinicopathological characteristics. Furthermore, the impact of TREK-1 on PCa cell proliferation and cycle was also investigated.

## MATERIALS AND METHODS

### Patient samples

A total of 100 patients with clinically localized PCa who received radical prostatectomy (RP) and extended pelvic lymphadenectomy in Fudan University, Shanghai Cancer Center (FUSCC) from January 2006 to July 2007 were included in this study. Those who had received neoadjuvant therapy were excluded from this study. Clinical and pathological information was obtained from electronic records and medical charts. The experimental protocols were approved by the Institutional Research Review Board at FUSCC, and written informed consent was obtained from all patients.

### Immunohistochemistry

Immunohistochemistry (IHC) was performed as previously described [15], using 5 μm tissue sections from formalin-fixed, paraffin-embedded PCa specimens and normal prostate tissues. In brief, after antigenicity retrieving, the sections were soaked with 3% H_2_O_2_-methanol for 15 min to block peroxidase activity, and incubated with 10% normal goat serum to block non-specific protein binding. Then the sections were incubated with primary antibody against *TREK-1* (1:100) (Santa Cruz Biotechnology, Dallas, TX), followed by anti-mouse/rabbit horseradish peroxidase-labeled antibody (Univ-bio, Shanghai, China) that was used as the second antibody. The expression of *TREK-1* was scored, considering the percentage of cells indicating a positive immunostaining profile and the intensity of the staining (from 0, 1+, 2+ and 3+).

### Cell culture

Human embryonic kidney 293T cells (HEK293T) and human PCa cell lines were purchased from the Institute of Cell Research of the Chinese Academy of Sciences (Shanghai, China). HEK293T, PC-3, DU145, LNCaP and 22RV1 cells were grown in DMEM medium, F-12K medium, MEM medium and RPMI1640 medium supplemented with 10% fetal bovine serum (Life Technology, Carlsbad, CA). Cells were incubated at 37ºC in 5% CO_2_.

### RNA extraction and quantitative real-time PCR

Total RNA was isolated from cultured cells using TRIzol reagent (Life Technology) according to the manufacturer's instructions. First strand cDNA was synthesized using the RevertAidTM First Strand cDNA Synthesis Kit (Life technology, Carlsbad, CA). Real-time PCR was performed using the Power SYBR Green PCR Master Mix (Life technology, Carlsbad, CA) in a 7900 HT Real-Time PCR system (Applied Biosystems, Foster City, CA). *β-actin* was used as an internal control. The primers sequences were as follows: *TREK-1*-forward: GTGACGCTGGCAACTTTGAT, reverse: AAACGAACAAACGCTGCTGT; *β-actin*-forward: AC CGAGCGCGGCTACAG, reverse: CTTAATGTCACGCA CGATTTCC.

### Vector construction and lentivirus production and infection

Short hairpin RNA (shRNA) for *TREK-1* was introduced into the PLKO.1 vector to generate PLKO.1-sh-TREK-1. The sequences of shRNA for *TREK-1* were as follows: shRNA1 F: 5′-CCGGGCGATCATATTCAAACACATACTCGAG TAT GTGTTTGAATATGATCGCTTTTTG-3′, R: 5′-AATTCA AAAAGCGATCATATTCAAACACATACTCGAGTATG TGTTTGAATATGATCGC-3′; shRNA2 F: 5′-CCGGCCA AAGTGGAAGATACGTTTACTCGAGTAAACGTATCT TCCACTTTGGTTTTTG-3′; R: 5′-AATTCAAAAACCA AAGTGGAAGATACGTTTACTCGAGTAAACGTATCT TCCACTTTGG-3′. PLKO.1-sh-TREK-1 was mixed with psPAX2 and PMD2-G and transfected into HEK293T cells using Lipofectamine 2000 reagent (Life Technology) according to the manufacturer's protocol. Forty-eight hours later, lentivirus was harvested and used to infect PCa cells. After infection, puromycin (2 μg/ml) was added into the medium to select stable infected cell clones for further experiments.

### Western blot

Total proteins were extracted using the CelLytic extraction kit containing protease inhibitors (Roche, Basel, Switzerland). Protein concentration was determined using the BCA Protein Assay reagent kit (Thermo Fisher Scientific, Waltham, MA, USA) according to the manufacturer's protocols. The proteins were separated using sodium dodecyl sulfate polyacrylamide gel electrophoresis, followed by transfer to polyvinylidene fluoride membranes. After blocking in 5% nonfat milk, the primary antibodies and anti-rabbit horseradish peroxidase second antibody (1:5000) were used to probe the target proteins. The bands were visualized using the ECL PlusWestern Blotting System (Thermo Fisher Scientific, Waltham, MA). GAPDH and β-tubulin were used as loading controls. The primary antibodies included the following: TREK-1 (1:1000), GAPDH (1:2000), β-tubulin (1:2000) (Santa Cruz Biotechnology, Dallas, TX), cyclin D1 (1:1000), cyclin E1 (1:500), CDK2 (1:500), p21 Cip1 (1:1000) and p27 Kip1 (1:1000) (Cell Signaling Technology, Boston, MA).

### Cell proliferation and colony formation assay

EdU (Ribobio, Guangzhou, China) and CCK-8 (Dojindo, Shanghai, China) assays were carried out to measure cell proliferation according to the manufacturer's instructions. Briefly, cells were cultured in EdU solution (1:5000) for 2 h, harvested and washed with phosphate-buffered saline (PBS) mixed with TritonX-100 (200:1). After staining using the Cell-Light EdU Apollo 643 *In Vitro* Flow Cytometry Kit (Ribobio, Guangzhou, China), cells were analyzed by flow cytometry (Beckman Coulter, Brea, CA, USA). For CCK-8 assay, cells were seeded in 96-well plates. After attaching to the bottom of the wells, cells were cultured in the medium mixed with CCK-8 (10:1) for 2 h. Absorbance was measured by a microplate reader at 450 nm.

For colony formation assay, cells were seeded in 60 mm plates at a concentration of 500 cells/well. After incubation in 5% CO_2_ at 37°C for 15 days, cells were fixed with 4% paraformaldehyde and stained with 0.5% crystal violet for 30 min. Colony numbers in each plate were counted.

### Cell cycle assay

Cell cycle was analyzed using flow cytometry. Briefly, cells were harvested and then washed twice in PBS. After fixing in 75% ice-cold ethanol overnight, cells were washed in PBS again and stained using propidium iodide (50 μg/ml) containing RNase for 30 min at room temperature. Cells were examined by flow cytometry.

### *In vivo* tumorigenicity

Animal experiments were performed under protocols approval by the Animal Studies Ethics Committee of FUSCC. Four-six-week-old male BALB/c nude mice were purchased from Shanghai SLAC Laboratory Animals Co., Ltd. (Shanghai, China). To establish tumor growth in nude mice, PC-3-Scr cells and PC-3-shRNA-TREK-1 cells were injected subcutaneously into either posterior flank of the same mouse. Using calipers, tumor sizes were measured at least three times weekly. The mice were euthanized with CO_2_ on the 28^th^ day. Tumor volume was calculated and tumor weight was measured after sacrificing.

### Statistical analyses

Comparison between two continuous variables was assessed by Student's t test. Difference of categorical variables was compared by chi-squared tests. Castration resistance free survival (CRFS) curves were drawn using the Kaplan–Meier method and compared by log-rank test. Cox's proportional hazard regression model was used to evaluate the relationship between CRFS and TREK-1 expression. A *P*-Value < 0.05 was considered statistically significant. All the statistical analyses were performed by SPSS version 20.0 software (IBM Corporation, Somers, NY, USA).

## RESULTS

### TREK-1 expression levels in PCa tissues

The patients’ characteristics are detailed in Table 1. The clinicopathological features of the total 100 patients with an average age of 72 years old (age range: 59–79 years old) with newly diagnosed, pathologically confirmed PCa were analyzed. Among the 100 total patients, 33 had received adjuvant hormonal therapy. During the follow-up of 2.0–93.3 months (median: 53.1 months), biochemical recurrence occurred in 52 patients. Additionally, two patients died of other diseases and only one patient died of PCa progression.

**Table 1:** Clinicopathological features of 100 PCa patients receiving RP

clinicopathological parameters	*n* (%)
**Age (y)**	59–79 (average 72)
**PSA (ng/ml)**	
<10	30 (30)
10–20	29 (29)
>20	41 (41)
**T stage**	
T2	87 (87)
T3	13 (13)
**N stage**	
N0	95 (95)
N1	5 (5)
**Gleason score**	
<7	29 (29)
7	41 (41)
>7	30 (30)

**Figure 1:**
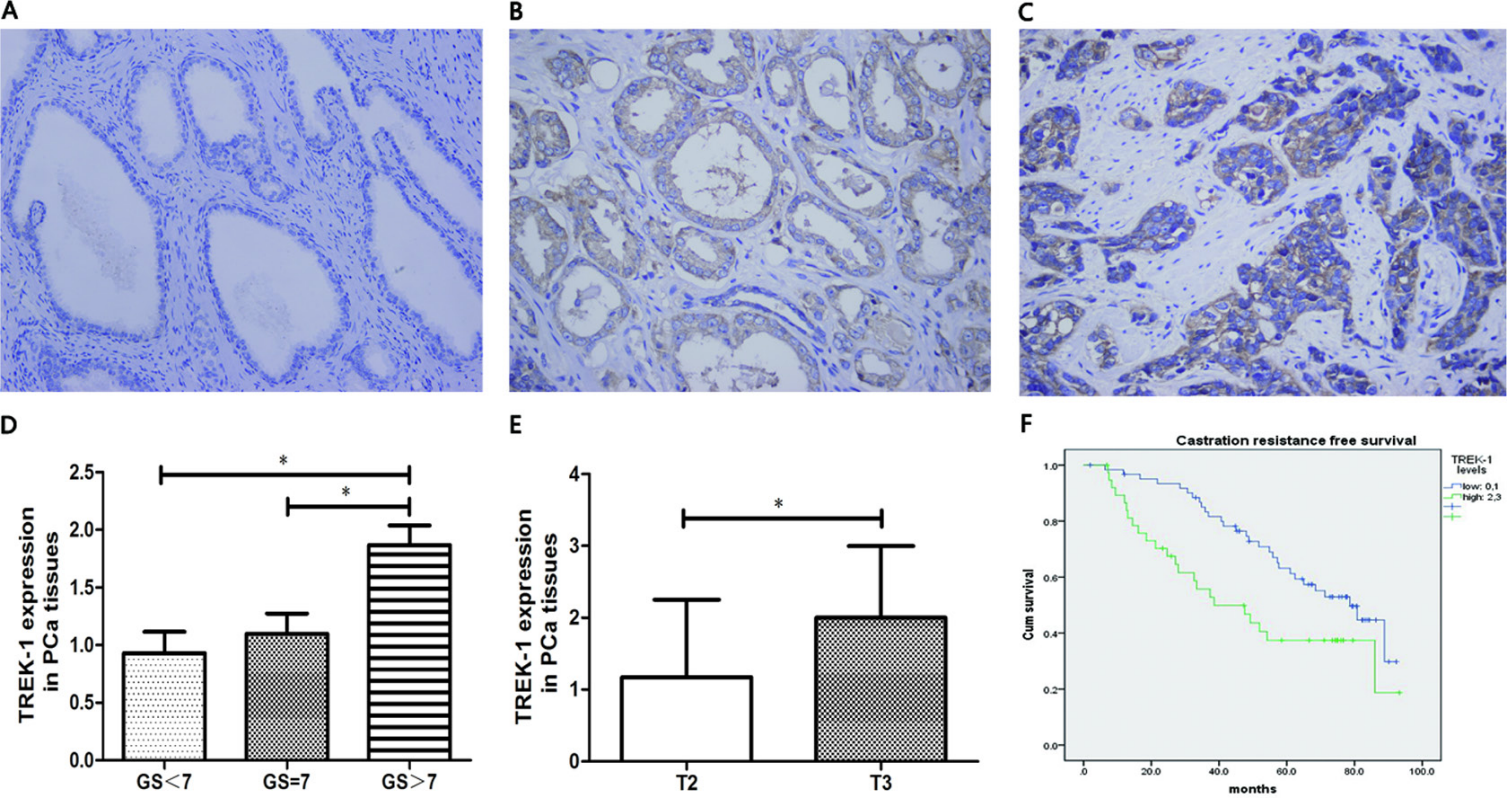
TREK-1 is overexpressed in PCa tissue and is associated with poor prognosis Immunohistochemistry staining showed overexpressed TREK-1 levels in PCa tissue **B, C.** compared with normal prostate tissue **A.** TREK-1 levels were positively associated with GS **D.** and T staging **E.** (*P < 0.05). **F.** Castration resistance free survival in patients with high levels of TREK-1 was significantly worse than the corresponding value in those with low levels of TREK-1.

We first examined TREK-1 protein expression in PCa tissues by IHC staining. TREK-1 staining was detected in 70 of 100 PCa specimens. Compared with normal prostate tissues, PCa tissues showed higher expression of TREK-1 levels (Figure 1A–1C). Furthermore, comparison of the levels of TREK-1 protein in PCa tissues with Gleason score (GS) grading and clinical stage revealed that TREK-1 levels were positively associated with GS and T staging (Figure 1D, 1E).

### Association of TREK-1 expression levels with biochemical recurrence of PCa patients receiving RP

Next, we examined the relationship between TREK-1 expression levels and biochemical recurrence in PCa patients receiving RP. All patients were divided into two groups based on TREK-1 staining levels: low level group (0 and 1+) and high level group (2+ and 3+). We found that CRFS in the high level group was significantly worse than in the low level group (median CRFS: 38.6 vs 78.6 months, respectively; *P* = 0.016) (Figure 1F). Multivariate analysis showed that, after adjusting for age, PSA levels, GS, T staging, lymph node involvement and history of adjuvant therapy, TREK-1 harbored a marginal predictive value for CRFS judgement (*P* = 0.061).

### Validation of TREK-1 knockdown in PCa cells

**Figure 2:**
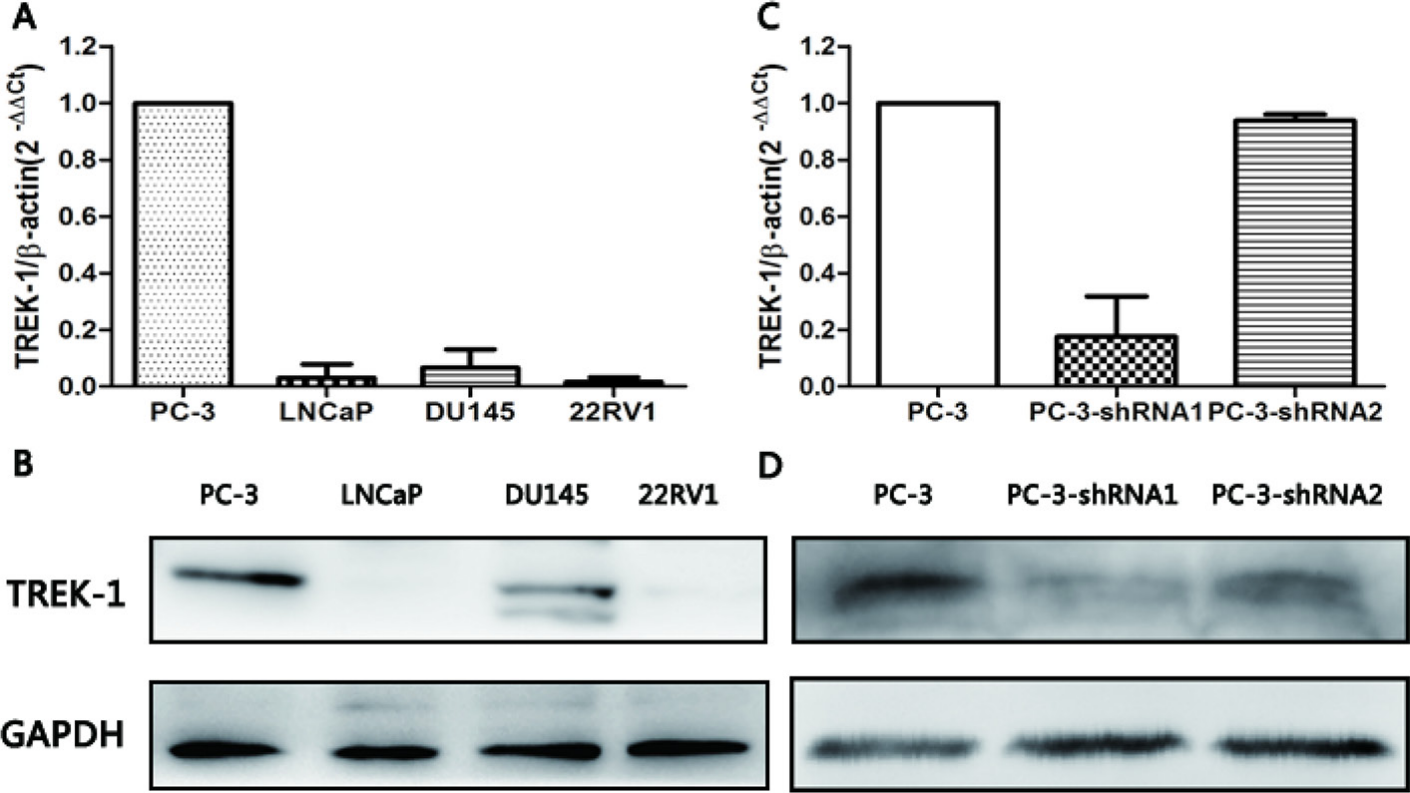
*TREK-1* expression profiles in human PCa cell lines and *TREK-1* knockdown PC-3 cells TREK-1 mRNA and protein expression was detected in four human PCa cell lines by RT-PCR **A.** and western blot **B.** Lower expression of TREK-1 mRNA and protein was detected in *TREK-1* knockdown PC-3 cells using RT-PCR **C.** and western blot **D.**

To investigate the biological function of TREK-1 in PCa, we measured the expression of TREK-1 mRNA and protein in four human PCa cell lines (PC-3, LNCaP, DU145 and 22Rv1) by quantitative real-time PCR and western blot. As shown in Figure 2A and 2B, relatively high expression of TREK-1 was detected in PC-3 cells at both mRNA and protein levels, while low expression was observed in the other three PCa cell lines. PC-3 cells were used for subsequent *TREK-1* knockdown experiments.

Using lentivirus-mediated shRNAs (*TREK-1*-sh1 and *TREK-1*-sh2), we knocked down *TREK-1* in PC-3 cells. Figure 2C and 2D showed the efficiency of shRNAs. PC-3 cells infected by *TREK-1*-sh1 exhibited particularly lower expression of *TREK-1* compared with controls, and were used in further experiments.

### Effects of TREK-1 knockdown on proliferation of PCa cells

**Figure 3:**
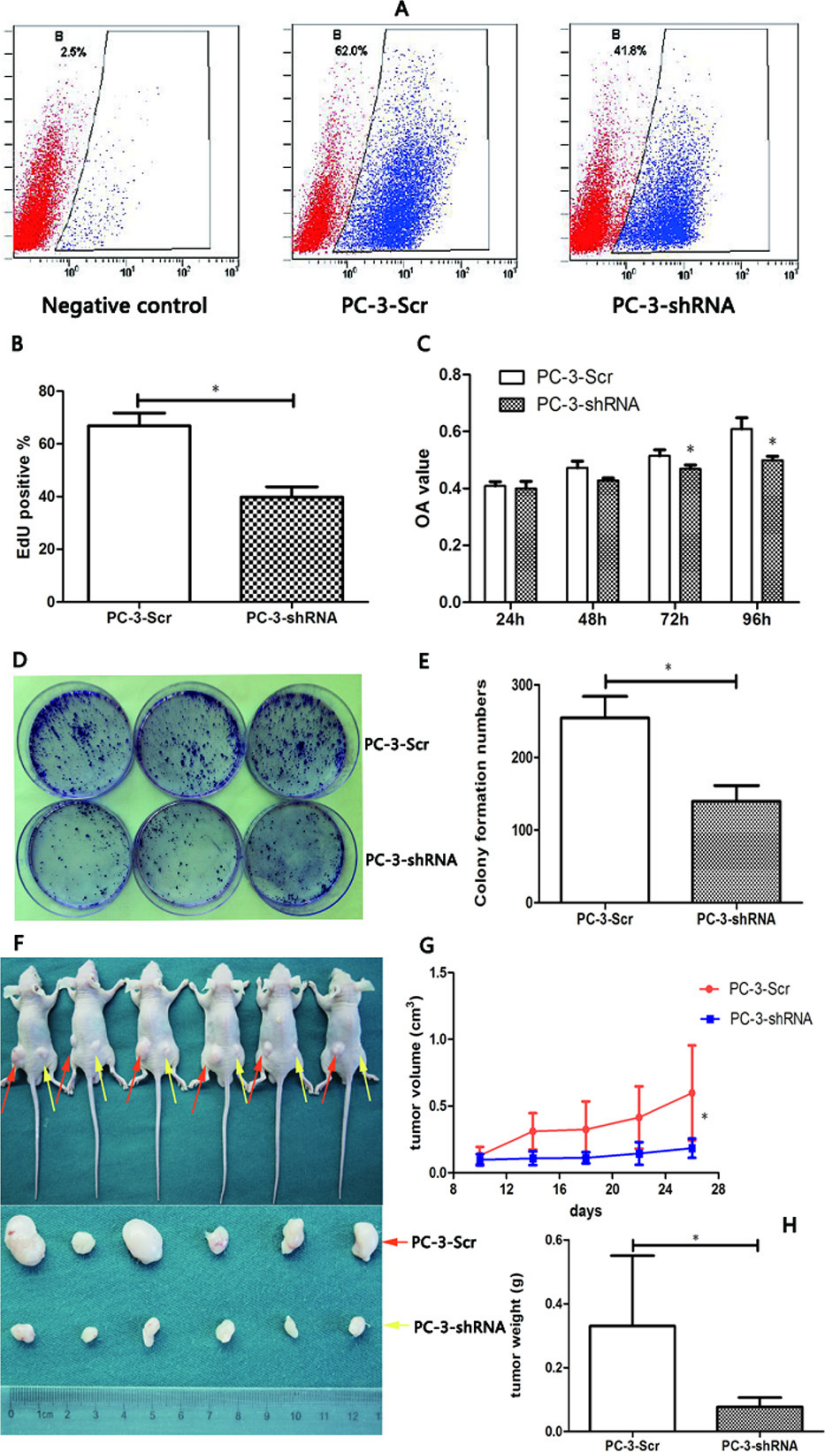
*TREK-1* knockdown significantly inhibits cell proliferation in PCa cells *in vitro* and *in vivo* **A, B.** Cell proliferation was detected in PC-3-Scr and PC-3-shRNA cells using EdU assay and analyzed by flow cytometry. **C.** A CCK-8 assay was performed to measure cell proliferation in *TREK-1* knockdown PC-3 cells and controls. Data represent the mean ± standard deviation of the optical density value detected at 450 nm from three independent experiments. **D, E.** Colony formation assays showed fewer colonies in *TREK-1* knockdown PCa cells. PC-3-Scr cells and *TREK-1*/PC-3-shRNA cells were injected into the left and right posterior flank of nude mice, respectively **F.** The tumor volume **G.** and mass **H.** in the PC-3-shRNA group were significantly lower than in the PC-3-Scr group (*P < 0.05).

To examine whether *TREK-1* knockdown affects cell proliferation of PCa cells *in vitro*, EdU, CCK-8 and colony formation assays were performed. Cell proliferation inhibition was observed in *TREK-1*/PC-3-shRNA cells compared with the control (Figure 3A–3C). Furthermore, *TREK-1*/PC-3-shRNA cells developed fewer colonies than the control at the 15^th^ day (Figure 3D, 3E)

To further determine the *in vivo* effect of TREK-1 in PCa cells, six subcutaneous tumor models were established using PC-3-Scr cells and *TREK-1*/PC-3-shRNA cells. After sacrificing at the 28^th^ day, both volume and mass were significantly lower in *TREK-1*/PC-3-shRNA tumors than those in controls (*P* < 0.05) (Figure 3F–3H). Taken together, our results demonstrated that *TREK-1* knockdown inhibited cell proliferation of PCa cells.

### Effect of TREK-1 knockdown on PCa cell cycle

**Figure 4:**
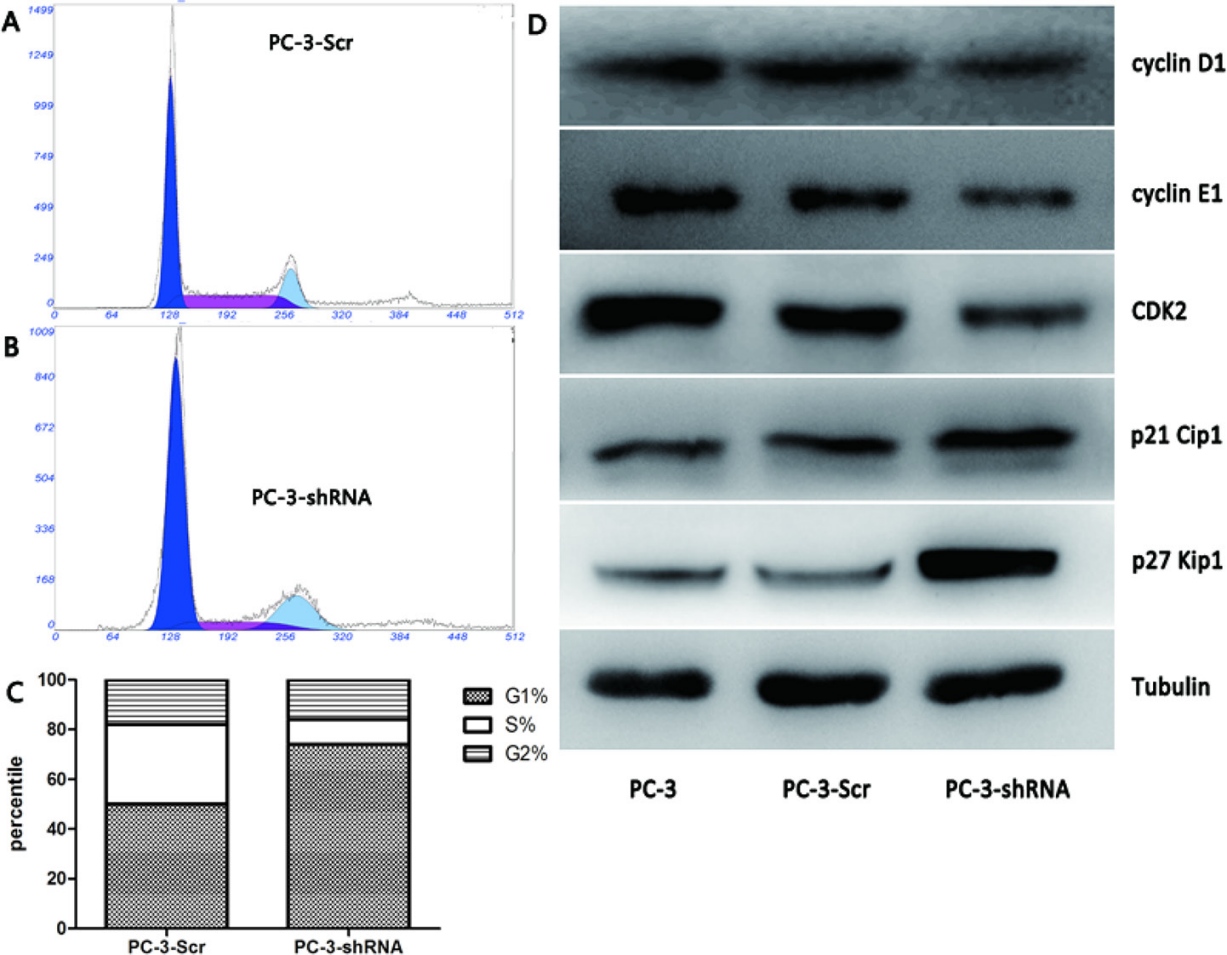
*TREK-1* knockdown induces G1/S phase cell cycle arrest in PCa cells The proportion of cells in G1 phase increased significantly in PC-3-shRNA cells compared with controls, while the proportion of cells in the S phase was notably less than the controls **A, B, C.** (*P < 0.05). **D.** Decreased cyclin D1, cyclin E1 and CDK2 levels, as well as elevated p21 Cip1 and p27 Kip1 levels were detected in TREK-1 knockdown PCa cells compared with controls.

As *TREK-1* knockdown exerts an inhibitory effect on PCa cell proliferation, we further investigated its role in the cell cycle. Compared with control cells, the proportion of PC-3-shRNA cells in G1 phase increased remarkably, while the proportion in S phase was reduced (Figure 4A–4C). The G1/S cell cycle arrest was accompanied by decreased cyclin D1, cyclin E1 and CDK2 levels, as well as elevated p21 Cip1 and p27 Kip1 levels (Figure 4D).

## DISCUSSION

K_2p_ channels maintain activity across the entire physiological voltage range. Their major role is to carry leak and background potassium currents that set the resting membrane potential and oppose depolarizing influences [16]. TREK-1 channels belong to the K_2p_ superfamily and are expressed mainly in the central and peripheral nervous system. *TREK-1* channels play an important role in neuroprotection against ischemia and epilepsy, and are possibly involved in the etiology of depression [17, 18]. Additionally, TREK-1 was found highly expressed in proliferative phase endometrium, which may in part be associated with increased cell division [19].

Although recent studies focusing on the role of potassium channels in human cancer have generated exciting findings [20], little information is known regarding the function of TREK-1 in carcinogenesis. Innamaa et al. investigated the expression and effects of TREK-1 in epithelial ovarian cancer [12]. They observed high expression of TREK-1 in ovarian cancer, yet no significant difference in survival was found between high and low IHC staining for TREK-1. The authors also found that TREK-1 blockers could inhibit cell proliferation of ovarian cancer cells through reducing early apoptosis and increasing late apoptosis. Voloshyna et al. reported that TREK-1 was overexpressed in prostatic intraepithelial neoplasia and PCa tissues in comparison with benign prostate tissues, and poor-differentiated PCa tissues indicated even higher expression of TREK-1 than well-differentiated ones [21]. Similar results were observed in our study. Moreover, we also analyzed the association of TREK-1 expression with biochemical recurrence in PCa patients receiving RP. We observed longer CRFS in patients with low levels of TREK-1 expression, suggesting that TREK-1 might play an important role in the initiation and development of PCa, and thus might be a potential biomarker in CRFS judgment of PCa. However, TREK-1 was not an independent predictor of biochemical recurrence of PCa. We speculate that the result might be in part ascribed to its positive association with GS and T staging. Accordingly, TREK-1, to a certain extent, may reflect the differentiation and progression of PCa.

Growing evidence has demonstrated the role of potassium channels in cell proliferation. DeCoursey et al. first linked potassium channels to the proliferative processes in T lymphocytes [22]. Since then, a variety of potassium channels have been implicated in the regulation of proliferation of colon cancer, hepatocarcinoma, lymphoma, breast cancer and bladder cancer cells [23–27]. In our study, we observed an inhibitory effect of TREK-1 knockdown on PCa cell proliferation, which was consistent with the findings of previous studies [19]. Using TREK-1 inhibitors, cell proliferation was notably suppressed in ovarian cancer cells and PCa cells [12, 21]. PCa cell proliferation could also be inhibited through overexpression of a dominant-negative TREK-1 mutant. Conversely, overexpression of TREK-1 was found to increase cell proliferation of normal prostate epithelium cells [21]. Furthermore, a higher density of expression of TREK-1 was observed in the ventricular subventricular zone during embryogenesis, which was consistent with its role in controlling cell proliferation [28]. The fact that TREK-1 contributes to cell proliferation might in part explain our finding that TREK-1 expression was positively associated with GS and T staging.

Although the underlying mechanisms regarding how potassium channels regulate cell proliferation are still a subject of debate, several events have been found to be controlled by these channels during cell proliferation, such as membrane potential, cell volume, Ca^2+^ signaling and cell cycle [20]. Two cell cycle blocks are often observed when potassium channels are suppressed: at G1 and at G2/M arrest with or without changes in the proportion of the S-phase cells [27]. Our results showed that TREK-1 knockdown induced G1/S cell cycle arrest in PC-3 cells. Meanwhile, a dramatic downregulation of cell cycle-related protein cyclin D1, cyclin E1 and CDK2, as well as upregulation of the cyclin-dependent kinases inhibitor p21 Cip1 and p27 Kip1 were observed. Additionally, the essential role of TREK-like currents in determining membrane potential has been reported [29], and Wonderlin et al. found that cell membrane potential was strongly correlated with G1 phase progression and cell proliferation [30]. Thus, the regulatory effect of *TREK-1* on the membrane potential might be closely associated with cell proliferation. Clearly, further research to elucidate the exact mechanisms by which *TREK-1* regulates cell cycle and proliferation is needed.

Our findings also give certain clues into TREK-1-targeted treatment of cancer. The anti-neoplastic property of curcumin, a TREK-1 blocker, has been found in various cancers, such as ovarian cancer, lung cancer, colorectal cancer, head and neck cancer [12, 31, 32]. Evidence from *TREK-1*−/− knockout mice demonstrated that *TREK-1* depletion could lead to a healthy fertile phenotype without morphological abnormalities [33]. Additionally, unlike many potassium channels blockers that may result in severe adverse effects such as cardiac arrhythmias, K_2p_ blockade has not been found to cause these side effects. Thus, *TREK-1* might in theory provide a new therapeutic target for PCa, which warrants further investigation.

Our study has several limitations that merit mentioning. First, our clinical information was analyzed retrospectively, which carries an intrinsic selection bias. Second, the follow-up of our study subjects was relatively short and we did not obtain more information, such as overall survival. Additionally, our results indicated that TREK-1 was not an independent predictor in CRFS judgement. Besides its inherent relationship with GS and T staging, our relatively smaller sample size might limit our statistical power. Hence, further prospective research in a large-scale patient groups with a long follow-up is needed.

In summary, our study found that *TREK-1* expression was upregulated in PCa compared with normal prostate tissues and showed a positive association with GS as well as T staging. Furthermore, *TREK-1* knockdown exhibited an inhibitory effect on PCa cell proliferation through G1/S cell cycle blockade. Although it is clear that TREK-1 is involved in PCa, further work is required to decipher more mechanisms underlying its roles in PCa progression and assess its potential value as a biomarker and/or therapeutic target.
